# Visual cueing and adaptive learning for teaching thyroid eye disease diagnostic assessment to ophthalmology residents

**DOI:** 10.3389/fmed.2026.1934529

**Published:** 2026-08-26

**Authors:** Jiajun Xie, Ji Shao, Changjun Wang, Xin Shi, Qi Miao, Chee Chew Yip, Juan Ye

**Affiliations:** 1 Eye Center of the Second Affiliated Hospital, Zhejiang University School of Medicine, Hangzhou, China; 2 Zhejiang Provincial Key Laboratory of Ophthalmology, Zhejiang University, Hangzhou, China; 3 Zhejiang Provincial Clinical Research Center for Eye Diseases, Zhejiang University, Hangzhou, China; 4 Zhejiang Provincial Engineering Institute on Eye Diseases, Zhejiang University, Hangzhou, China; 5 Ophthalmology & Visual Sciences Department, Khoo Teck Puat Hospital, National Healthcare Group, Singapore, Singapore; 6 Yong Loo Lin School of Medicine, National University of Singapore, Singapore, Singapore; 7 Lee Kong Chian School of Medicine, Nanyang Technological University, Singapore, Singapore

**Keywords:** adaptive learning, medical education, ophthalmology resident, static case review, thyroid eye disease, visual cueing

## Abstract

**Background:**

Thyroid eye disease (TED) diagnosis requires residents to interpret subtle clinical and imaging findings for activity and severity assessment. Guided by Cognitive Load Theory, visual cueing may support attention to diagnostically relevant features, whereas adaptive learning may tailor practice to learner performance. This pilot study compared visual cueing, adaptive learning, and traditional static case review for TED diagnostic training.

**Methods:**

Forty-five ophthalmology residents were randomised equally to static case review (SCR), an adaptive learning system (ALS), or a visual cueing atlas (VCA). Following a 45-minute intervention, residents completed immediate and two-week delayed tests. The primary outcome was immediate overall diagnostic accuracy. Secondary outcomes were Clinical Activity Score (CAS) accuracy, severity-grading accuracy, delayed-test performance (2 weeks post-intervention), and performance decline from immediate to delayed testing. Educational materials were validated by ten expert ophthalmologists. Item-level content validity indices and the scale-level content validity index were calculated from expert ratings.

**Results:**

All residents completed the study. Expert validation showed excellent content validity for the educational materials, with the Item-level Content Validity Index for all components and the overall Scale-level Content Validity Index both reaching 1.00. At the immediate post-test, VCA achieved higher CAS accuracy than SCR (84.7% vs. 64.7%, *p* < 0.001). ALS and VCA achieved higher severity-grading accuracy than SCR (83.3% and 78.7% vs. 66.7%; *p* = 0.004 and *p* = 0.025, respectively), and both achieved higher overall diagnostic accuracy than SCR (66.0% each vs. 51.3%; *p* = 0.014 and *p* = 0.008, respectively). At two weeks, ALS showed higher severity-grading accuracy than SCR (74.0% vs. 56.0%, *p* < 0.001), while both ALS and VCA maintained higher overall diagnostic accuracy than SCR (54.0% and 52.7% vs. 41.3%; *p* = 0.014 and *p* = 0.008, respectively).

**Conclusion:**

In this pilot study, visual cueing was associated with higher immediate CAS accuracy, whereas adaptive learning was associated with higher severity-grading accuracy on the two-week delayed test, relative to static case review. These approaches may have complementary roles in TED diagnostic education. Larger studies using equivalent instructional exposure, matched assessment forms, and longer follow-up are needed before a staged hybrid approach is recommended.

## Introduction

Thyroid eye disease (TED) is a complex autoimmune orbital disorder and the most common benign orbital condition with a prevalence of 0.1% to 0.3% (1, 2), affecting 25%–49% of patients with Graves' disease (3, 4). It has a myriad of presentations including eyelid retraction, proptosis, conjunctival inflammation, diplopia, and exposure-related corneal disease (5). In severe cases, sight-threatening optic neuropathy and diplopia result in diminished quality of life (6, 7). Accurate assessment is essential because clinical decisions depend on distinguishing disease activity from severity and identifying features requiring urgent intervention. In routine practice, this requires interpretation of multiple visual and clinical cues, including eyelid and conjunctival changes, proptosis, ocular motility, optic nerve status, anterior-segment photographs, and orbital imaging.

For ophthalmology residents, assessment of TED is challenging because the Clinical Activity Score (CAS) and disease-severity classifications require recognition and integration of subtle, variably expressed signs. Features such as conjunctival injection, chemosis, caruncular oedema, eyelid swelling, and soft-tissue changes may be difficult to identify consistently, even among experienced clinicians (8, 9). Inter-observer variability in TED assessment further illustrates the difficulty of applying standardised criteria reliably in clinical practice (9). Although case-based learning is widely used to contextualise clinical knowledge and support application to practice (10, 11), static case review (SCR) is a fixed form of case-based learning in which learners review clinical information, images, and expert classifications without additional scaffolding to guide visual attention or tailor practice to learner performance. SCR may therefore provide limited support in directing novice attention or regulating task difficulty.

This educational challenge can be understood through Cognitive Load Theory, which posits that learning is constrained by working memory capacity (12, 13). For novice learners, TED diagnosis imposes a high intrinsic cognitive load because they must integrate clinical history, photographs, imaging findings, activity criteria, and severity classifications. Unguided interpretation of complex images may add extraneous cognitive load when learners are uncertain which signs require attention. Instructional strategies that provide visual guidance or adapt task difficulty may therefore support diagnostic learning within this theoretical framework.

Visual cueing and adaptive learning may offer complementary instructional approaches that are theoretically relevant to cognitive load management during TED diagnostic training. Visual cueing uses explicit annotations or visual scaffolds on medical images to guide learners' visual search behaviours (14, 15). Based on the Cognitive Load Theory, such guidance has been proposed to reduce inefficient visual search and extraneous processing, potentially preserving working-memory resources for interpreting disease activity and severity (15–17). Adaptive learning, conversely, is an instructional approach that dynamically adjusts the delivery of educational content based on real-time learner interactions (18). Within this framework, adaptive learning may help manage task-related cognitive demands by matching case progression to learner performance through scaffolding (19): Presenting similar cases after errors may provide further practice with the relevant diagnostic distinction, whereas progression after correct responses introduces case variability without unnecessary repetition. Together, these approaches may support diagnostic learning across image-based clinical cases. However, their relative effects have rarely been compared directly in ophthalmology residency education.

This pilot study compared three instructional formats for TED diagnostic training: traditional SCR, an adaptive learning system (ALS), and a visual cueing atlas (VCA). Guided by Cognitive Load Theory, we hypothesised that VCA would preferentially support immediate recognition of activity-related clinical signs through visual guidance, whereas ALS would support the application and short-term retention of severity-grading criteria through performance-contingent practice. The primary objective was to compare immediate post-intervention overall diagnostic accuracy across the three modalities. Secondary objectives were to compare CAS accuracy, severity-grading accuracy, two-week delayed-test performance, and performance decline from immediate to delayed testing.

## Materials and methods

### Study design

This was a single-centre, pilot randomised educational study comparing three instructional methods (ALS, VCA, and SCR) for teaching thyroid eye disease (TED) diagnostic assessment to ophthalmology residents. The study evaluated the diagnostic performance and short-term retention of knowledge among the residents taught using the three methods. As this was an exploratory study of novel instructional strategies within an available resident cohort, no formal *a priori* sample-size calculation was performed.

The primary outcome was *overall diagnostic accuracy*, defined as the proportion of cases where residents correctly identified both the CAS and its severity grading, and was measured by the immediate post-test. The secondary outcome measures were twofold: (1) *short-term knowledge retention*, assessed via the delayed post-test at week 2 for CAS accuracy, severity-grading accuracy, and overall diagnostic accuracy. (2) *skill decay*, defined as the absolute percentage-point decline from the immediate post-test to the delayed post-test.

### Participants

Participants comprised 45 ophthalmology residents recruited from the Eye Center of the Second Affiliated Hospital, Zhejiang University School of Medicine. Eligible participants were ophthalmology residents actively undergoing clinical training who provided written informed consent prior to enrolment. Participation was voluntary, and residents could withdraw from the study at any time without academic or professional consequences. Individuals who were not actively engaged in clinical training, were unable to complete the full 45-minute educational intervention, or failed to complete all evaluation points were excluded from the analysis.

### Randomisation and allocation concealment

Participants were randomly assigned in a 1:1:1 ratio to the SCR, ALS, or VCA group. The randomisation sequence was generated using a computer-generated random-number procedure. Each participant was assigned a random number, and the final group allocation was determined by sorting these numbers by magnitude. Allocation was concealed using sequentially numbered, opaque, sealed envelopes that were opened only after enrolment and baseline preparation were completed.

Because of the nature of the educational interventions, participants and supervising instructors could not be blinded to the learning format after allocation. However, all diagnostic assessments were administered through the online platform and scored automatically against predefined expert answers, reducing assessor-related bias. Participants were instructed to complete the learning sessions independently and were explicitly prohibited from communicating with peers or sharing online training materials during the study period.

### Educational interventions

All interventions were delivered through a secure, self-developed online learning platform. Each group was allocated 45 min for the assigned learning session. SCR and VCA participants each reviewed five prespecified cases selected from the 20-case TED database. In the ALS group, cases were dynamically selected from the same 20-case database according to individual performance. Therefore, the number and sequence of cases could vary within the standardised 45-minute learning period. The three instructional formats were as follows.

For the SCR group, the residents reviewed five complete static TED cases. Each case included the clinical history, unaltered clinical and imaging materials, and the final expert classification of disease activity and severity. This format represented conventional case-based review.

For the ALS group, the residents interacted with an automated, rule-based case-delivery system. For each presented case, participants were required to assess both disease activity using CAS and disease severity according to established criteria. If both components were answered correctly, the system advanced to another case from the database. If the CAS assessment was incorrect, the system presented a case with similar activity-related features to reinforce recognition of activity-related features. If the severity grading was incorrect, the system presented a case with similar severity-related features to support targeted practice. If both CAS and severity grading were incorrect, the pre-defined CAS-related pathway took priority. Cases were presented without replacement within each learning session, so the same case was not repeated. Case presentation continued according to these pre-defined rules until the end of the 45-minute session.

For the VCA group, the residents reviewed a modified image atlas in which key pathological signs on clinical photographs and relevant anatomical features on CT images were explicitly marked. The visual images were designed to direct attention to diagnostically relevant features, such as conjunctival injection, chemosis, caruncular oedema, eyelid swelling, proptosis-related changes, extraocular muscle enlargement, and other features relevant to TED activity and severity assessment.

### Development and validation of educational content

A standardised database of 20 representative clinical TED cases was retrospectively developed from our institutional electronic medical records. Each case included a brief clinical history, anterior-segment photographs, and orbital computed tomography (CT) images. All identifying facial features were masked or cropped to protect patient confidentiality. The same case database was used to develop the three instructional formats.

Before implementation, all educational cases, visual annotations, and adaptive-delivery logic were evaluated by a panel of 10 senior ophthalmologists. To achieve a high standard for residency training, the materials underwent an iterative refinement process. In the first round, the experts independently rated the materials using a four-point rubric across five domains: accuracy, completeness, clarity, appropriateness, and clinical-reasoning quality. For items that did not meet the predefined criterion or for which disagreement arose regarding a specific clinical feature, the experts provided the rationale for their assessments. The relevant materials were then revised accordingly and independently re-evaluated in a second round. The reported I-CVI and S-CVI/Ave values were calculated from the final post-revision ratings.

### Study procedures

**Figure 1:**
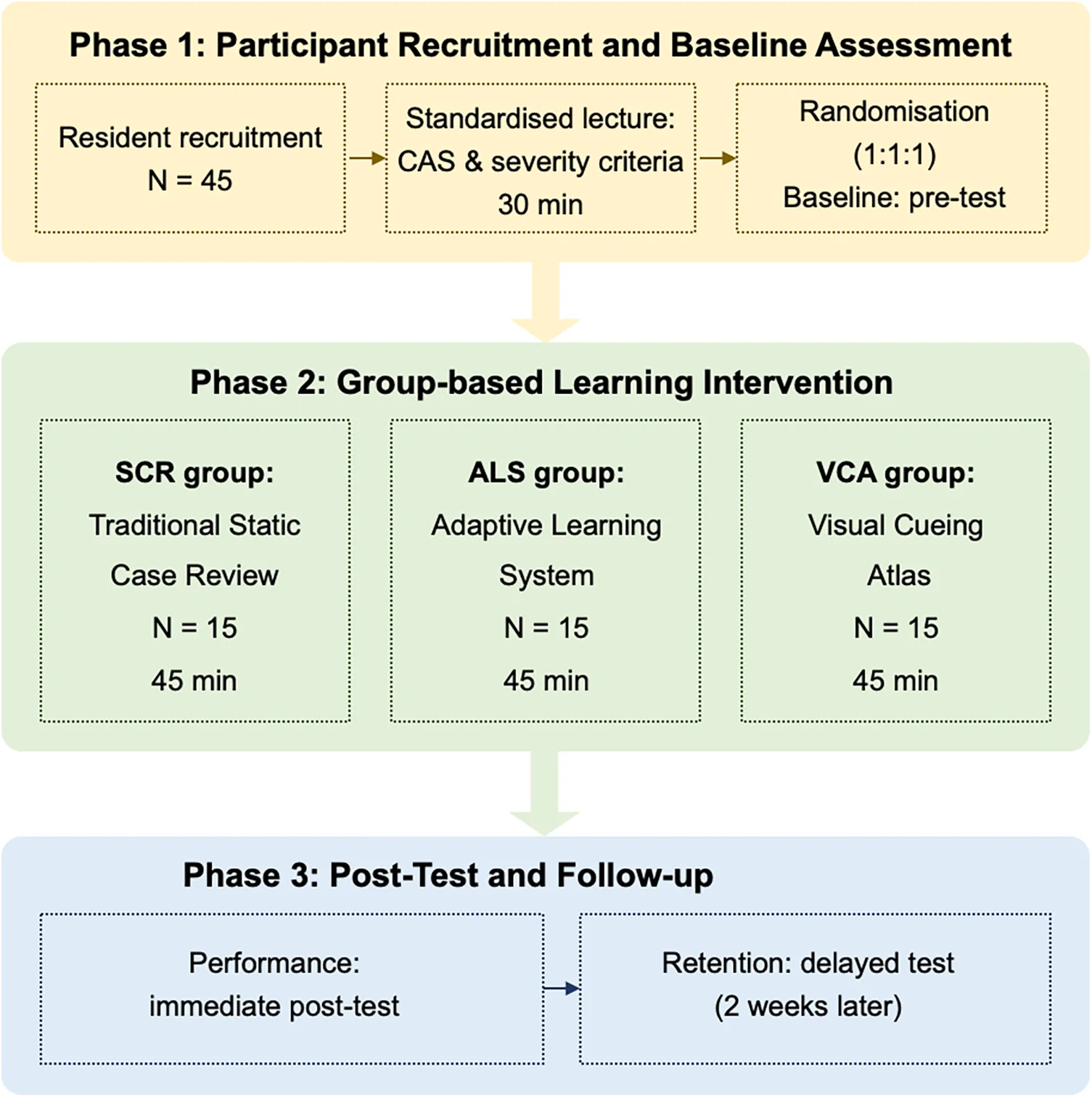
Flow diagram of the pilot randomised controlled trial. The flowchart illustrates the enrolment, randomisation, intervention allocation, and follow-up phases of the 45 participating ophthalmology residents. Participants were evenly randomised (*n* = 15 per group) into three instructional cohorts: SCR, Static case review; ALS, Adaptive learning system; and VCA, Visual cueing atlas.

The steps of the study are shown in Figure 1. Prior to the educational intervention, all participants completed a standard 30-minute preparatory learning on TED diagnostic criteria, specifically including the Clinical Activity Score (CAS) and disease severity classifications recommended by the European Group on Graves' Orbitopathy (20) and the American Thyroid Association/European Thyroid Association guidelines (3, 21). Following baseline preparation, participants were randomised to the three study groups and completed a baseline pre-test. They then undertook their assigned 45-minute educational intervention, which was supervised by experienced clinical instructors to ensure protocol adherence, with no additional diagnostic teaching provided beyond the assigned intervention materials. Immediately after the intervention, participants completed the immediate post-test for CAS accuracy, severity-grading accuracy, and overall diagnostic accuracy. To evaluate short-term knowledge retention, a delayed post-test was subsequently conducted two weeks later for the same three pre-test measures.

### Instruments

The standardised diagnostic tests for the pre-test, immediate post-test, and delayed post-test were developed and validated by a panel of senior oculoplastic specialists. Each assessment consisted of 10 novel clinical cases that were mutually exclusive, ensuring no overlap across testing time points or duplication of the training materials to evaluate true diagnostic transfer. These assessments were administered through a self-directed online educational platform where residents independently reviewed the clinical history, photographs, and imaging data to determine the disease activity and severity. The system subsequently auto-calculated diagnostic accuracy against predefined standard expert answers across three core domains. Specifically, CAS accuracy was calculated as the percentage of cases where the resident correctly identified the exact CAS, while severity-grading accuracy reflected the correct classification into the appropriate severity category according to EUGOGO criteria. As defined above, the overall diagnostic accuracy was scored as a binary case-level outcome requiring both CAS and severity grading to be correct; a resident had to correctly identify both the CAS and the severity grading simultaneously for a given case to receive a correct score, whereas an error in either component resulted in a score of zero. This composite outcome was selected because TED assessment requires concurrent evaluation of both disease activity and severity, which provide distinct but complementary information. Binary case-level scoring ensured that correct performance in one component did not compensate for an incorrect classification in the other. CAS accuracy and severity-grading accuracy were therefore also analysed separately as secondary outcomes to allow component-specific interpretation.

### Data analysis

Descriptive statistics were used to summarise participant demographics. Categorical variables were described as frequencies and percentages, while continuous variables were presented as mean ± standard deviation (SD).

Validation of the educational materials was assessed using the content validity index (CVI) based on the ratings provided by the 10-expert panel. The Item-level CVI (I-CVI) was calculated as the proportion of experts assigning a rating of 3 (Good) or 4 (Excellent) on a four-point scale across five domains: accuracy, completeness, clarity, appropriateness, and clinical-reasoning quality. The overall Scale-level CVI (S-CVI/Ave) was calculated as the mean of the I-CVI values to determine the overall content validity.

To evaluate the effects of the interventions on diagnostic performance across the pre-test, immediate post-test, and delayed post-test, repeated-measures ANOVA was used to analyse the main effects of time, group, and the time-by-group interaction. Differences in performance decline between groups were examined using one-way analysis of variance. All *post-hoc* pairwise comparisons were adjusted using Bonferroni correction. Statistical significance was defined as *p* < 0.05. Residual normality was assessed using the Shapiro–Wilk test, and homogeneity of variance across groups was assessed using Levene's test at each time point. No significant violations were identified. Sphericity was not assumed, and Geisser-Greenhouse correction was applied to within-subject effects and time-by-group interactions. Standardised effect sizes were additionally reported to complement significance testing. Partial eta squared (*η*p²) was calculated for repeated-measures ANOVA effects, and Hedges' g was used for pairwise between-group comparisons because of the relatively small group sizes. Corresponding 95% confidence intervals were reported where applicable. Statistical analyses and graph generation were performed using GraphPad Prism version 8.0 (GraphPad Software, San Diego, CA, United States).

### Ethical considerations

The study was conducted in accordance with the principles of the Declaration of Helsinki and received approval from the Institutional Review Board of the Second Affiliated Hospital, Zhejiang University School of Medicine (Approval No. 2025 [1006]) prior to study initiation. Written informed consent was obtained from all participants before enrolment. Study data were anonymised, stored on secure institutional servers, and analysed in aggregate form.

## Results

### Participant characteristics

A total of 45 ophthalmology residents were enrolled, randomised, and completed all phases of the study. Participants were evenly assigned to the SCR, ALS, and VCA groups, with 15 residents in each group. Baseline demographic and educational characteristics were comparable across the three groups as shown in Table 1. The mean ages of participants in the SCR, ALS, and VCA groups were 26.60 ± 2.47, 27.33 ± 2.58, and 28.27 ± 1.87 years respectively, with no statistically significant difference among groups (*p* = 0.158). The proportions of female residents were 73.3%, 66.7%, and 53.3% in the SCR, ALS, and VCA groups, respectively (*p* = 0.507). The distribution of residency training level was also comparable, with senior residents comprising 20.0%, 40.0%, and 33.3% of the SCR, ALS, and VCA groups, respectively (*p* = 0.484). These findings indicated no significant baseline differences among the groups before the intervention.

**Table 1:** Baseline demographic and educational characteristics of the study participants.

Characteristics	SCR (*n* = 15)	ALS (*n* = 15)	VCA (*n* = 15)	*p* value
Age (years), mean ± SD	26.60 ± 2.47	27.33 ± 2.58	28.27 ± 1.87	0.158
Gender, *n* (%)				0.507
Male	4 (26.7%)	5 (33.3%)	7 (46.7%)	
Female	11 (73.3%)	10 (66.7%)	8 (53.3%)	
Residency year, *n* (%)				0.484
Junior (Year 1–2)	12 (80.0%)	9 (60.0%)	10 (66.7%)	
Senior (Year 3)	3 (20.0%)	6 (40.0%)	5 (33.3%)	

SCR, Static case review; ALS, Adaptive learning system; VCA, Visual cueing atlas; SD, standard deviation.

### Expert validation of educational content

Prior to the educational intervention, the training materials were validated by a panel of 10 senior specialists. Following the two-round expert review and revision process, all 10 experts rated the final materials as good or excellent (ratings of 3 or 4) across all five evaluated domains. The Item-level Content Validity Index (I-CVI) for all components and the overall Scale-level Content Validity Index (S-CVI/Ave) both achieved a score of 1.00. The mean evaluation scores ranged from 3.87 to 4.00 (out of 4.0), with a median score of 4 across all domains. These I-CVI and S-CVI/Ave values were calculated from the final post-revision ratings (Table 2).

**Table 2:** Expert evaluation of the educational cases, visual annotations, and personalised delivery logic for thyroid eye disease.

Evaluation Domain	Mean Score (SD)	Median	I-CVI	Interpretation
Accuracy	4.00 (0.00)	4	1.00	Fully aligned with current clinical guidelines
Completeness	3.88 (0.33)	4	1.00	Comprehensive coverage of key clinical elements
Clarity	3.87 (0.34)	4	1.00	Clear visual cues and textual explanations with minimal ambiguity
Appropriateness	3.95 (0.23)	4	1.00	Clinically safe and contextually appropriate for residency training
Clinical Reasoning Quality	3.98 (0.14)	4	1.00	High-level clinical reasoning, differential diagnosis, and decision-making.
Overall (S-CVI/Ave)	—	—	1.00	Excellent content validity

Scores were provided by a panel of 10 senior ophthalmologists based on a four-point rubric (1 = poor, 2 = fair, 3 = good, 4 = excellent).

**I-CVI** (Item-level Content Validity Index) represents the proportion of experts rating each domain ≥ 3.

**S-CVI/Ave** (Scale-level Content Validity Index) represents the average I-CVI across all evaluated domains.

### Instructional exposure

All participants completed the assigned 45-minute learning session. Participants in the SCR and VCA groups each reviewed five fixed cases. Platform records showed that ALS participants completed a mean of 7.47 ± 1.19 cases, with a median of 7 (IQR 7–8) and a range of 6–10 cases during the same 45-minute period. Thus, although learning duration was standardised across groups, the number of cases encountered was greater and more variable in the ALS group.

### Primary outcome: immediate overall diagnostic accuracy

**Figure 2:**
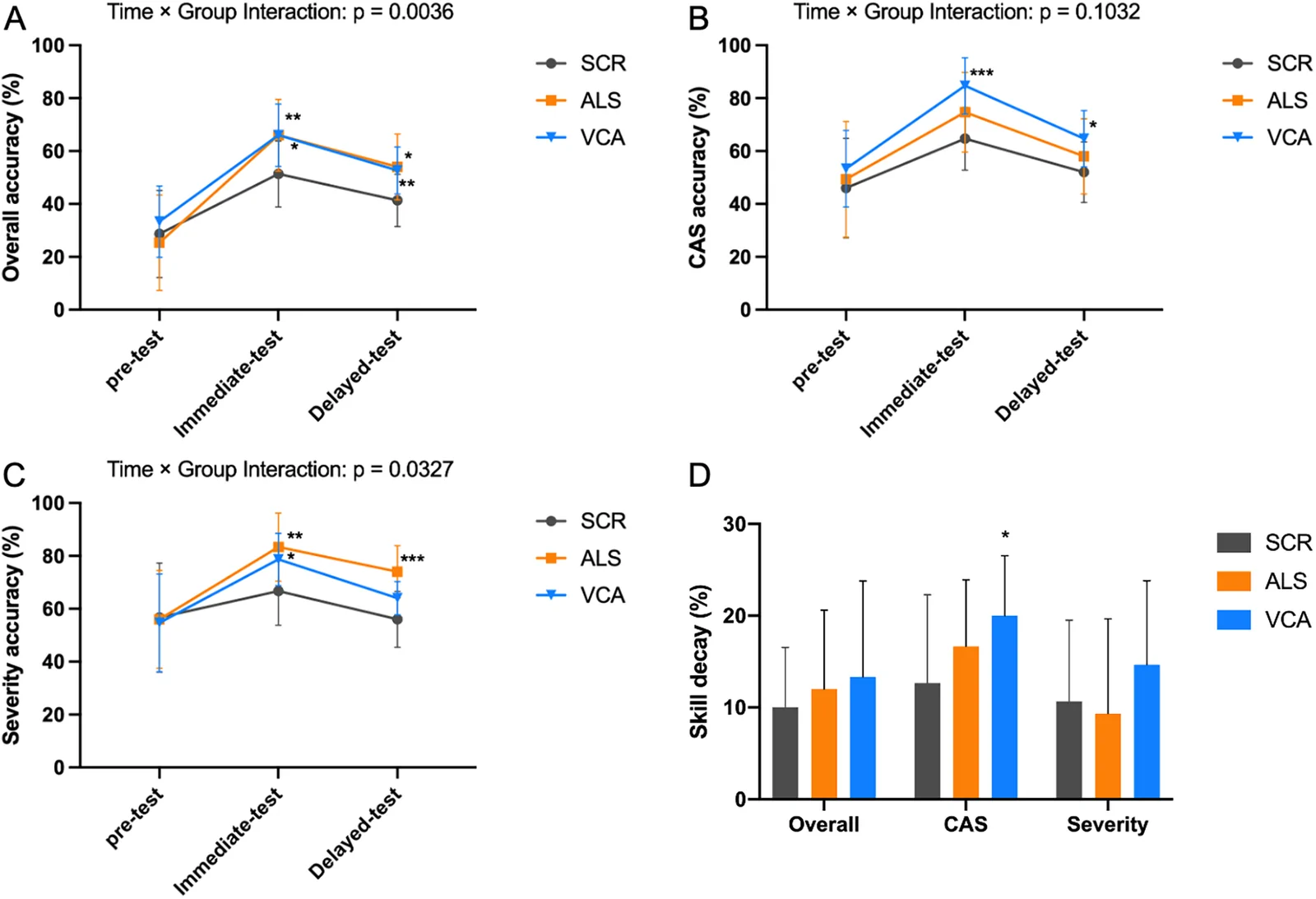
Longitudinal performance and skill retention analysis. **(A)** Overall accuracy (primary outcome) across the three groups. **(B,C)** Secondary outcomes including CAS accuracy **(B)** and severity grading accuracy **(C)** across the three groups. **(D)** Skill decay from the immediate to the delayed post-test across the three diagnostic domains. Data are presented as mea*n* ± SD. SCR, Static case review; ALS, Adaptive learning system; VCA, Visual cueing atlas. *p < 0.05, **p < 0.01, ***p < 0.001 vs. SCR group. Geisser-Greenhouse-corrected time × group interaction effects are shown for the longitudinal performance panels **(A–C)**.

Baseline diagnostic assessments confirmed that the three groups were comparable before the intervention, with one-way ANOVA revealing no statistically significant differences in overall diagnostic accuracy (*p* = 0.4006), CAS accuracy (*p* = 0.5631), or severity-grading accuracy (*p* = 0.9587). For overall diagnostic accuracy, repeated-measures ANOVA revealed a significant time-by-group interaction after Geisser-Greenhouse correction [F(2.864, 60.15) = 5.145, *p* = 0.0036] (Figure 2A). Bonferroni-adjusted *post-hoc* comparisons showed that the ALS group achieved an overall accuracy of 66.0% ± 13.5%, which was significantly higher than the SCR group (51.3% ± 12.5%, *p* = 0.0136). The VCA group also achieved an accuracy of 66.0% ± 11.8%, significantly higher than that in the SCR group (*p* = 0.0078). No statistically significant difference was observed between the ALS and VCA groups (*p* > 0.9999).

At the two-week delayed post-test, both the ALS (54.0% ± 12.4%) and VCA groups (52.7% ± 8.8%) maintained overall accuracy scores that were significantly higher than the SCR group (41.3% ± 9.9%; *p* = 0.0140 and *p* = 0.0079, respectively). Detailed diagnostic performance data are presented in Table 3. Effect sizes for the repeated-measures analysis and pairwise comparisons are provided in Supplementary Tables S1 and S2, respectively.

**Table 3:** Comparison of primary and secondary diagnostic outcomes across three educational strategies.

Outcomes & time points	SCR (*n* = 15)	ALS (*n* = 15)	VCA (*n* = 15)	Mean Diff. (95% CI) vs. SCR	Adjusted *p* value*
Primary Outcome: Overall Accuracy (%)					
Pre-test (omnibus *p* = 0.4006)	28.7 ± 16.4	25.3 ± 18.1	33.3 ± 13.5	ALS: −3.4 (−19.4 to 12.7) VCA: 4.6 (−9.3 to 18.7)	ALS: >0.9999 VCA: >0.9999
Immediate post-test	51.3 ± 12.5	66.0 ± 13.5*	66.0 ± 11.8**	ALS: 14.7 (2.6 to 26.8) VCA: 14.7 (3.4 to 26.0)	ALS: 0.0136 VCA: 0.0078
Delayed post-test	41.3 ± 9.9	54.0 ± 12.4*	52.7 ± 8.8**	ALS: 12.7 (2.2 to 23.1) VCA: 11.3 (2.6 to 20.1)	ALS: 0.0140 VCA: 0.0079
Secondary Outcome: CAS Accuracy (%)					
Pre-test (omnibus *p* = 0.5631)	46.0 ± 18.8	49.3 ± 21.9	53.3 ± 14.5	ALS: 3.3 (−15.7 to 22.3) VCA: 7.3 (−8.3 to 23.0)	ALS: >0.9999 VCA: 0.7270
Immediate post-test	64.7 ± 11.9	74.7 ± 15.1	84.7 ± 10.6***	ALS: 10.0 (−2.7 to 22.7) VCA: 20.0 (9.5 to 30.5)	ALS: 0.1607 VCA: 0.0001
Delayed post-test	52.0 ± 11.5	58.0 ± 14.2	64.7 ± 10.6*	ALS: 6.0 (−6.1 to 18.1) VCA: 12.7 (2.4 to 22.9)	ALS: 0.6440 VCA: 0.0119
Secondary Outcome: Severity Grading (%)					
Pre-test (omnibus *p* = 0.9587)	56.7 ± 20.6	56.0 ± 18.4	54.7 ± 18.5	ALS: −0.7 (−18.9 to 17.5) VCA: −2.0 (−20.2 to 16.2)	ALS: >0.9999 VCA: >0.9999
Immediate post-test	66.7 ± 12.9	83.3 ± 12.9**	78.7 ± 9.9*	ALS: 16.6 (4.7 to 28.7) VCA: 12.0 (1.3 to 22.7)	ALS: 0.0043 VCA: 0.0248
Delayed post-test	56.0 ± 10.6	74.0 ± 9.9*** #	64.0 ± 6.3	ALS: 18.0 (8.5 to 27.5) VCA: 8.0 (−0.2 to 16.2)	ALS: 0.0001 VCA: 0.0578

Data are expressed as mean ± SD. SCR, static case review; ALS, adaptive learning system; VCA, visual cueing atlas; CI, confidence interval. Pairwise mean differences, 95% CIs, and *p* values were adjusted using Bonferroni correction after repeated-measures ANOVA. Mean differences are presented as intervention group minus SCR.

* *p* < 0.05.

** *p* < 0.01.

*** *p* < 0.001 vs. SCR group.

# *p* < 0.01 vs. VCA group.

### CAS accuracy

For CAS accuracy, repeated-measures ANOVA showed no statistically significant time-by-group interaction after Geisser-Greenhouse correction [F(2.893, 60.75) = 2.168, *p* = 0.1032] (Figure 2B). At the immediate post-test, the VCA group achieved an accuracy of 84.7% ± 10.6%, which was significantly higher than the 64.7% ± 11.9% recorded in the SCR group (*p* = 0.0001). The difference between the VCA group and the ALS group (74.7% ± 15.1%) did not reach statistical significance (*p* = 0.1368). At the two-week delayed post-test, the VCA group retained an accuracy of 64.7% ± 10.6%, remaining significantly higher than the SCR group (52.0% ± 11.5%, *p* = 0.0119). Full pairwise comparisons are shown in Table 3, with corresponding effect sizes provided in Supplementary Tables S1, S2.

### Severity grading accuracy

For severity grading assessment, a significant time-by-group interaction was observed after Geisser-Greenhouse correction [F(2.530, 53.12) = 3.343, *p* = 0.0327] (Figure 2C). At the immediate post-test, both the ALS group (83.3% ± 12.9%, *p* = 0.0043) and the VCA group (78.7% ± 9.9%, *p* = 0.0248) scored significantly higher than the SCR group (66.7% ± 12.9%). The difference between the ALS and VCA groups was not significant (*p* = 0.8302). At the delayed post-test, the ALS group recorded an accuracy of 74.0% ± 9.9%, which remained significantly higher than the SCR group (56.0% ± 10.6%, *p* = 0.0001). Full pairwise comparisons are shown in Table 3, with corresponding effect sizes provided in Supplementary Tables S1, S2.

### Performance decline from immediate to delayed testing

Performance decline was calculated as the absolute percentage-point decline from the immediate to the delayed post-test and showed varying patterns across the diagnostic domains. For overall accuracy decay, the declines for the SCR, ALS, and VCA groups were 10.0% ± 6.5%, 12.0% ± 8.6%, and 13.3% ± 10.5%, respectively, with no statistically significant differences among the three groups (*p* = 0.5761). In the domain of CAS, a significant difference in decay was observed (*p* = 0.0494). Bonferroni-adjusted *post-hoc* comparisons revealed that the VCA group exhibited the largest decline (20.0% ± 6.5%), which was significantly higher than the 12.7% ± 9.6% decay in the SCR group (*p* = 0.0447). The decay observed in the ALS group (16.7% ± 7.2%) did not significantly differ from the SCR group (*p* = 0.5199). For severity grading, the absolute decays were 10.7% ± 8.8%, 9.3% ± 10.3%, and 14.7% ± 9.2% for the SCR, ALS, and VCA groups, respectively, with no significant differences across groups (*p* = 0.2857). Detailed datasets for skill decay metrics are presented in Table 4 and Figure 2D.

**Table 4:** Comparison of skill decay across three educational strategies.

Diagnostic Domains	SCR (*n* = 15)	ALS (*n* = 15)	VCA (*n* = 15)	Omnibus *p* value	Mean Diff. (95% CI) vs. SCR	Adjusted *p* value*
Overall Accuracy Decay (%)	10.0 ± 6.5	12.0 ± 8.6	13.3 ± 10.5	0.5761	ALS: 2.0 (−5.9 to 9.9) VCA: 3.3 (−4.6 to 11.3)	ALS: >0.9999 VCA: 0.8989
CAS Accuracy Decay (%)	12.7 ± 9.6	16.7 ± 7.2	20.0 ± 6.5*	0.0494	ALS: 4.0 (−3.2 to 11.2) VCA: 7.3 (0.1 to 14.5)	ALS: 0.5199 VCA: 0.0447
Severity Grading Decay (%)	10.7 ± 8.8	9.3 ± 10.3	14.7 ± 9.2	0.2857	ALS: −1.3 (−9.9 to 7.3) VCA: 4.0 (−4.6 to 12.6)	ALS: >0.9999 VCA: 0.7605

Data are expressed as mean ± SD. Skill decay is defined as the absolute percentage-point decline from the immediate to the delayed post-test. SCR, Static case review; ALS, Adaptive learning system; VCA, Visual cueing atlas. Omnibus *p* values were calculated using one-way ANOVA. Pairwise mean differences, 95% confidence intervals (CIs), and *p* values were adjusted using Bonferroni corrections following one-way ANOVA.

* *p* < 0.05 vs. SCR group.

## Discussion

### Principal findings

This pilot randomised trial compared traditional SCR, VCA, and ALS for teaching TED diagnostic assessment to ophthalmology residents. Both active instructional formats were associated with higher immediate overall diagnostic accuracy and severity-grading accuracy than static case review. VCA was associated with the highest immediate accuracy in CAS assessment, whereas ALS showed the highest severity-grading accuracy at the two-week delayed test among the three groups. At the delayed test, both active formats maintained higher overall diagnostic accuracy than SCR. Taken together, these findings suggest that visual cueing may be particularly helpful for early recognition of activity-related signs, whereas adaptive learning may be more relevant to repeated application and short-term knowledge retention of severity-grading criteria. These findings should be interpreted as preliminary because of the small pilot sample and potential variation in instructional exposure.

Beyond statistical significance, the magnitude of the observed differences may also be educationally relevant. Several significant between-group differences were approximately 12–20 percentage points, corresponding to roughly one to two additional correctly classified cases in a 10-case assessment. These differences suggest a potentially meaningful improvement in residents' performance on structured TED diagnostic tasks. However, no established minimal educationally important difference is available for this assessment, and the present study evaluated image-based diagnostic performance rather than workplace-based clinical competence. The findings should therefore be interpreted as evidence of improved assessment performance rather than direct evidence of improved clinical decision-making or patient care.

### Interpretation through cognitive load theory

The findings can be interpreted through Cognitive Load Theory, which distinguishes the intrinsic complexity of a task from extraneous demands imposed by its presentation (12, 13). TED assessment requires novice learners to integrate clinical history, external photographs, orbital imaging, CAS criteria, and disease-severity classifications. Unguided visual interpretation may also add extraneous cognitive load when learners are uncertain which signs are diagnostically relevant.

The immediate advantage of VCA in CAS assessment may reflect the visual guidance provided for subtle inflammatory signs, such as conjunctival injection, chemosis, caruncular oedema, and eyelid swelling. By highlighting diagnostically relevant features, visual cueing may have supported attention to clinically relevant information. This interpretation is consistent with evidence that expert diagnostic performance is associated with more efficient visual attention patterns in image-based tasks (14, 22, 23). However, cognitive load and gaze behaviour were not measured; therefore, reduced visual-search demands and cognitive load remain theoretically-informed explanations rather than demonstrated mechanisms.

The delayed severity-grading advantage observed with ALS may reflect performance-contingent case sequencing. Participants who answered incorrectly were presented with cases with similar activity- or severity-related features, whereas correct responses led to new cases. Such sequencing provided repeated opportunities to retrieve and apply diagnostic criteria and may have supported targeted practice in areas of difficulty. This interpretation is consistent with adaptive learning and retrieval-practice literature, which suggests that targeted practice and active recall may improve retention relative to passive review (18, 24, 25). However, ALS participants completed more cases on average than participants in the fixed-case groups (7.47 vs. 5 cases), despite the same 45-minute learning period. Therefore, the observed advantage may reflect both performance-contingent sequencing and greater practice exposure and cannot be attributed to adaptive sequencing alone.

However, these explanations should be interpreted cautiously. This study did not directly measure cognitive load, gaze behaviour, visual-search efficiency, or schema formation. Therefore, the proposed mechanisms remain theoretically-informed interpretations rather than directly demonstrated learning processes.

### Comparison with existing literature

The immediate CAS advantage observed with VCA is broadly consistent with the literature showing that visual guidance can support early learning in image-based diagnostic tasks. Novice learners may lack stable perceptual schemas and attend less efficiently to diagnostically relevant visual information than experts (14, 22). Eye-tracking studies similarly suggest that expertise is associated with more efficient visual attention patterns during image interpretation (22, 23). In this context, visual cueing may have helped residents identify subtle inflammatory features of TED.

The greater absolute decline in CAS accuracy in the VCA group should be interpreted cautiously. Because VCA achieved the highest immediate CAS performance, it also had greater scope for decline at the delayed assessment. Nevertheless, the pattern is consistent with the principle that instructional scaffolds may support early learning but should be progressively faded to promote independent performance (26). For TED training, visual annotations may assist initial recognition of key signs, but subsequent unaided practice is likely needed to support transfer to new cases without visual cues.

The ALS findings are also consistent with the literature on adaptive learning and retrieval practice. Adaptive systems can adjust case sequencing or content exposure according to learner performance, thereby directing practice towards areas of difficulty and potentially improving learning efficiency (18, 27). Repeated testing and active retrieval may further support retention more effectively than passive review (24, 25). In the present study, the higher delayed severity-grading accuracy in the ALS group is consistent with the possibility that error-contingent case practice provided repeated opportunities to apply severity criteria. However, because ALS participants completed a greater and more variable number of cases than participants in the fixed-case groups, the observed effect may reflect both adaptive sequencing and greater practice exposure.

This study extends previous work by directly comparing visual cueing and adaptive learning with SCR in ophthalmology residency training. Much of the existing image-based education literature has focused on radiology (14), pathology (28), and dermatology (29). The present findings provide preliminary, domain-specific evidence that visual cueing and adaptive learning may support different aspects of diagnostic learning in TED.

### Educational implications

The findings suggest that SCR alone may provide limited support for novice learners in visually complex diagnostic tasks. Although it presents expert information and final classifications, it may not adequately guide attention towards subtle signs or provide targeted practice after diagnostic errors.

VCA may be useful during the early phase of TED diagnostic training, when residents are learning to identify activity-related signs such as eyelid swelling, conjunctival injection, chemosis, and caruncular oedema. By making these features explicit, visual annotations may help learners develop initial perceptual familiarity with TED activity assessment.

ALS may be more useful when learners need repeated practice applying diagnostic criteria across varied presentations. TED severity grading requires integration of multiple findings and consistent use of classification rules. Performance-contingent case sequencing may allow learners to revisit specific areas of difficulty while progressing after correct responses.

Together, these findings support further evaluation of a staged instructional model in which visual cueing is introduced early, gradually faded, and followed by adaptive case-based practice requiring increasingly independent diagnostic decisions. Such an approach may also be relevant to other visually intensive areas of ophthalmology, including retinal optical coherence tomography interpretation, optic-disc assessment, corneal disease evaluation, and orbital imaging. However, this model remains a hypothesis for future study rather than an established curricular recommendation.

### Strengths

This study has several strengths. First, the randomised design allowed direct comparison of two theoretically distinct instructional strategies with traditional SCR within the same educational context. Second, the study focused on TED, a clinically important and visually complex condition in which residents may have limited exposure and difficulty integrating activity and severity criteria. Third, the educational cases, visual annotations, and adaptive-delivery logic underwent expert review before implementation, supporting the content validity and clinical appropriateness of the instructional materials. Fourth, the assessments were administered through an online platform with automated scoring against predefined expert answers, limiting assessor-related bias. The training cases and the three assessment sets were mutually exclusive, which was intended to limit direct recall effects and to allow evaluation of diagnostic performance on novel cases. Finally, inclusion of both immediate and two-week delayed assessments provided preliminary evidence of short-term retention beyond immediate post-intervention gains.

### Limitations

Several limitations should be considered when interpreting these findings. This study was a single-centre pilot study with a small convenience sample and no formal *a priori* sample-size calculation. The limited sample size reduces the statistical precision and stability of the effect estimates, particularly for secondary outcomes and multiple pairwise comparisons. Therefore, the findings should be interpreted as exploratory and require confirmation in larger studies. Prior TED-specific teaching or clinical exposure before enrolment was not systematically recorded, and residual differences in baseline experience cannot be excluded. The findings may therefore not be generalisable to residents with different levels of prior TED exposure or to other training settings.

Although learning time was standardised across groups, instructional exposure was not equivalent. ALS participants completed a mean of 7.47 ± 1.19 cases (range 6–10), whereas SCR and VCA participants each reviewed five fixed cases. The observed ALS effect may therefore reflect both performance-contingent sequencing and greater practice exposure, and the independent contribution of adaptive sequencing cannot be determined from the present study. Detailed platform-interaction metrics, including time spent per case and branch-specific activity, were not retained, limiting further comparison of engagement and effective practice exposure across interventions. In addition, participants and supervising instructors could not be blinded to the visibly different learning formats, which may have influenced engagement.

The training and assessment cases were mutually exclusive, reducing direct recall effects; however, formal psychometric equivalence of the three assessment sets was not established. The study also assessed image-based diagnostic performance over only two weeks rather than longer-term retention, workplace-based clinical competence, or patient outcomes. Accordingly, improvements in assessment performance should not be interpreted as direct evidence of improved clinical competence or patient care. Finally, cognitive load, visual-search behaviour, eye movements, learner engagement, and mental effort were not measured directly. The proposed mechanisms should therefore be regarded as theoretically-informed interpretations rather than experimentally demonstrated learning processes.

### Future directions

Future multicentre studies with larger and more diverse resident cohorts across different educational settings and with varying levels of prior TED-specific teaching and clinical exposure are needed to confirm these preliminary findings and permit adequately powered subgroup analyses. Such studies should standardise instructional exposure across groups, use matched and validated assessment sets, and include longer follow-up to determine whether gains in diagnostic performance are sustained.

Further research should evaluate a staged curriculum in which early visual cueing is progressively faded and followed by adaptive case-based practice requiring independent diagnostic decisions. Objective process measures, including eye-tracking, cognitive-load ratings, response-time data, and platform-use analytics, may clarify how visual cueing and adaptive sequencing influence diagnostic learning. Workplace-based assessments should also be incorporated to determine whether improvements in image-based testing transfer to clinical TED assessment and management.

## Conclusion

In this pilot study, visual cueing was associated with higher immediate CAS accuracy, whereas adaptive learning was associated with higher two-week severity-grading accuracy than static case review. These approaches may support complementary aspects of TED diagnostic learning. Larger studies with equivalent instructional exposure, longer follow-up, and workplace-based outcomes are needed before a staged combined approach is recommended.

## Data Availability

The raw data supporting the conclusions of this article will be made available by the authors, without undue reservation.
