# Supplementary material for: Visual cueing and adaptive learning for teaching thyroid eye disease diagnostic assessment to ophthalmology residents
Source: Front Med (Lausanne). 2026 Aug 26;13:1934529. doi: 10.3389/fmed.2026.1934529 (PMC13509029; doi:10.3389/fmed.2026.1934529)
Supplement: Supplementary file 1 [file Table1.docx]

**Supplementary Table S1 Effect sizes for time-by-group interactions**

| **Outcome** | **F (GG-corrected df)** | **p value** | **Partial ηp²** |
| --- | --- | --- | --- |
| Overall diagnostic accuracy | F(2.864, 60.15) = 5.145 | **0.0036** | **0.197** |
| CAS accuracy | F(2.893, 60.75) = 2.168 | 0.1032 | **0.094** |
| Severity grading accuracy | F(2.530, 53.12) = 3.343 | **0.0327** | **0.137** |

**Notes:** SCR, static case review; ALS, adaptive learning system; VCA, visual cueing atlas; CAS, Clinical Activity Score; GG, Geisser-Greenhouse; ηp², partial eta squared.

**Supplementary Table S2 Hedges’ g for pairwise comparisons**

| **Outcomes & Time points** | **Hedges’ g vs. SCR** | **Adjusted p value** |
| --- | --- | --- |
| **Overall diagnostic accuracy** | | |
| - Pre-test | ALS: -0.19  VCA: 0.30 | ALS: >0.9999  VCA: >0.9999 |
| - Immediate post-test | ALS: 1.10  VCA: 1.17 | ALS: **0.0136**  VCA: **0.0078** |
| - Delayed post-test | ALS: 1.10  VCA: 1.17 | **ALS: 0.0140**  VCA: 0.0079 |
| **CAS accuracy** | | |
| - Pre-test | ALS: 0.16  VCA: 0.43 | ALS: >0.9999  VCA: 0.7270 |
| - Immediate post-test | ALS: 0.72  VCA: 1.73 | ALS: 0.1607  VCA: 0.0001 |
| - Delayed post-test | ALS: 0.45  VCA: 1.12 | ALS: 0.6440  VCA: 0.0119 |
| **Severity-grading accuracy** | | |
| - Pre-test | ALS: -0.03  VCA: -0.10 | ALS: >0.9999  VCA: >0.9999 |
| - Immediate post-test | ALS: 1.26  VCA: 1.02 | **ALS: 0.0043**  VCA: 0.0248 |
| - Delayed post-test | ALS: 1.72  VCA: 0.90 | **ALS: 0.0001**  VCA: 0.0578 |

**Notes:** SCR, static case review; ALS, adaptive learning system; VCA, visual cueing atlas; CAS, Clinical Activity Score; Hedges’ g was calculated using the pooled standard deviation with a small-sample correction. Positive Hedges’ g values indicate higher performance in the intervention group relative to SCR, whereas negative values indicate lower performance. p values are Bonferroni-adjusted pairwise comparisons corresponding to those reported in Table 3.
